# The Relationship Between Sports Club Participation, Physical Activity, and Health Behaviors Among Older Korean Adults

**DOI:** 10.3390/healthcare12232411

**Published:** 2024-12-01

**Authors:** Jeonga Kwon, Jusun Jang

**Affiliations:** 1 Department of Elementary Education, College of First, Korea National University of Education, Cheongju-si 28173, Republic of Korea; gilddong1234555@knue.ac.kr; 2 Department of Sports for All, College of Arts, Cheongju University, Cheongju-si 28503, Republic of Korea

**Keywords:** health behaviors, older adults, perceived health status, physical activity, sports clubs

## Abstract

Background: Participation in physical activity can improve the physical, mental, and social health of older adults, with greater benefits observed when they join sports clubs or groups rather than exercising alone. Despite the numerous advantages of older adults’ participation in sports clubs, research on this topic remains scarce. This study investigated the association between participation in sports clubs and weekly physical activity, perceived health status, and health behaviors (adequate rest and sleep and regular meals and nutritional supplementation) among Korean older adults. Methods: Data on 5146 individuals aged 60 years or older, collected from the 2022–2023 Korea National Lifestyle and Sports Survey, were analyzed using frequency, chi-square, and multivariate logistic regression analyses. Results: Significant differences were found in sex, age, weekly physical activity, perceived health status, adequate rest and sleep, regular meals and nutritional supplementation, use of exercise prescription and consultation services, participation in lifestyle physical education classes, and cessation of drinking and smoking based on participation in sports clubs. Sports club participation increased the frequency of weekly physical activity and improved perceptions of health status in older adults. The average odds ratios (ORs) for weekly physical activity were 6.667 (95% confidence interval CI = 4.316–10.297; *p* < 0.001), 5.237 (95% CI = 3.372–8.133; *p* < 0.001), and 3.042 (95% CI = 1.864–4.966; *p* < 0.001) for 1–2, 3–4, and 5 or more days of participation, respectively. The average ORs were 0.559 (95% CI = 0.264–1.183; *p* = 0.128) for inadequate rest and sleep, and 1.272 (95% CI = 0.555–1.694; *p* = 0.100) for adequate rest and sleep. Average ORs were 0.976 (95% CI = 0.497–1.915; *p* = 0.943) for irregular meals and nutritional supplementation, and 1.028 (95% CI 0.770–1.373; *p* = 0.851) for regular meals and nutritional supplementation. However, sports club participation was not significantly associated with sufficient rest and sleep or regular meals and nutritional supplementation. Conclusions: Older adults need support to join sports clubs, which can promote their physical activity and health.

## 1. Introduction

In 2015, the global population of individuals aged 65 years and older was 617 million, with the percentage of older people projected to rise significantly over the next 35 years [1]. The fastest-growing age group will be those over 80, expected to reach 447 million by 2050, tripling the current figure [1]. In Korea, the proportion of people aged 65 years and over increased from 7.2% in 2000 to 14.9% in 2019. By 2025, this population is expected to exceed 20.3%, making South Korea a super-aged society [2]. This transition will have occurred in 25 years, a much faster rate compared to other countries [3]. Particularly, Korean older adults often seek emotional and financial support from the state and their families. Nonetheless, with the increase in unmarried and single-person households, individualization has increased, necessitating that older adults voluntarily take responsibility for their livelihood and health [4]. Thus, the Korean government has established various policies to address the growing older adult population, such as the creation of jobs and healthcare projects [5]. The rapid transition to a super-aged society, as seen in South Korea compared to France’s 155 years and Japan’s 35 years, highlights the seriousness of the problem [3]. Unpreparedness for this demographic shift has led to demographic imbalances, slower economic growth, health problems, and intergenerational conflicts [6].

Aging is an inevitable natural phenomenon [7]. Living an active disease-free life in old age can bring immense happiness [7]. However, increasing the longevity and quality of life of older adults remains a challenge [8]. Recognizing aging as a social issue [9], it is important to find ways to lead a healthier and more active life in old age. Physical activity can improve the quality of life of older adults. Accordingly, Korea has been promoting sports policies to ensure a healthy and enjoyable retirement through sports support tailored to older adults [10]. For example, various physical activity programs are conducted in places with many older adults, such as welfare, senior, and community centers [10]. Continued participation in physical activity among older adults is associated with a reduced risk of cardiovascular diseases, decreased disability and frailty, beneficial metabolic profile, improved independence, and better quality of life [11]. Furthermore, the positive effects of physical activity on mood can persist over time [12,13]. Moreover, older adults exhibit greater mental health benefits from physical activity than young adults [14]. Evidently, older adults’ participation in physical activity significantly affects their health [15], with stronger effects observed when they join sports clubs or groups rather than participate alone [16,17,18].

Specifically, Stolarz et al. [16] found that participation in senior clubs positively impacted the functional status, quality of life, and physical activity levels of older adults. Ma et al. [17] proved that older adults who regularly participated in soccer clubs had lower body mass index, higher muscular endurance, and higher total body endurance, which positively impacted their physical health. Furthermore, Watts et al. [18] observed an association between being a member of a sports club and reduced frailty, mediated by increased physical activity. This is because sports clubs provide an easy way for older adults to maintain social relationships, engage in physical activity, and feel satisfied and happy [19]. Older adults who engage in physical activity in sports clubs tend to remain active for a long duration and enjoy excellent quality of life and physical function [16]. Additionally, they have lower fat percentage and body mass index than those who do not participate [17]. Considering that physical activity levels decrease with age [19], joining sports clubs can increase older adults’ physical activity. Despite the numerous advantages of older adults’ participation in sports clubs, research on this topic remains scarce. Kwon and Lee [20] explored the sports club culture among older adults, focusing on constraints related to participation, satisfaction, immersion, and sustainability. Therefore, this study examined the association between participation in sports clubs and weekly physical activity, perceived health status, and health behaviors among Korean older adults. Previous studies indicated that weekly physical activity, perceived health status, and health behaviors were factors directly and indirectly related to health [11,12,13,14,15,16,17,18,19,20]. These variables contribute to the promotion and maintenance of health in older adults.

Hence, we designed a study based on Kelley-Gillespie’s Integrated Conceptual Model of Quality of Life for Older Adults [21]. This conceptual model describes the impact on the quality of life in older adults across six well-being-related domains: social, physical, psychological, cognitive, spiritual, and environmental [21]. Each domain is divided into several dimensions to facilitate the measurement of the quality of life [21]. Given the association between physical activity/health behaviors and the quality of life among older adults, we adopted this model. The research questions were as follows: First, “Is there an association between sports club participation and weekly physical activity among Korean older adults?” Second, “Is there an association between sports club participation and perceived health status among Korean older adults?” And third, “Is there an association between sports club participation and health behaviors among Korean older adults?” We aimed to provide foundational data for research on sports club participation among older adults. The findings of this study will motivate older adults to participate in sports clubs, contributing to the development of a healthier society.

## 2. Materials and Methods

### 2.1. Study Population and Data Collection

This study used data from the 2022–2023 Korea National Lifestyle Sports Survey. Since 2014, this sample survey has been conducted annually nationwide to analyze sports participation and facility utilization among citizens. The questionnaire used in this study, first produced in 1991 after expert consultation and literature review, was later updated through expert meetings [22]. The questionnaire is refined annually. Just before the survey, experts gather to review the validity and reliability of this questionnaire. Additionally, experts have validated and reviewed the 2022–2023 version. Specifically, the questionnaire development was as follows: First, the objectives, methods, and direction of the study were established by a council of experts. Moreover, the relevant literature was examined to understand the concept, necessity, purpose, current status, and challenges of life and physical education, as well as the current status and prospects of life sports in foreign countries. Second, a research model was created by integrating findings from previous studies and expert opinions. Third, reliability and validity were analyzed through data collection, review, and analysis. Fourth, the questionnaires were revised and refined after an expert meeting before the start of the 2022 and 2023 surveys, with adjustments made to the survey content. The questionnaire contained questions related to health awareness, regular physical activity, alcohol and smoking cessation, and adequate rest and sleep.

We collected data from 18,000 individuals aged 10 years and older. The study analyzed data from 5146 individuals aged over 60 years, excluding non-respondents. The sample included 2451 males, 2695 females, 2691 in their 60s, 2341 in their 70s, and 114 aged 80 or older. This was carried out based on the United Nations Population Division’s definition of older adults as those over the age of 60 [23]. The survey dataset, excluding personally identifiable information, is posted on the website of the Korean Institute of Sports Science (https://www.sports.re.kr/front/board/bs/boardList.do?board_seq=74&menu_seq=865; accessed on 4 September 2024). We collected the data solely for research purposes. Because the dataset used did not include personally identifiable information, such as telephone numbers, home addresses, names, and social security numbers, ethical approval was not required for this study. Nonetheless, this study was conducted according to the principles outlined in the Declaration of Helsinki. Informed consent was obtained from all participants. Specifically, the background and necessity of the survey and the national approval statistics were explained through the survey document. Participants were asked to contact us by phone if they had any questions about the survey. Consent regarding personal information was obtained in the questionnaire. Only those who agreed to participate responded to the questionnaire.

### 2.2. Independent and Dependent Variables

The independent variable was sports club participation, assessed by the question “Have you joined a sports club, and do you participate in it?” The response options were “I have joined a sports club, and I participate in it”, “I have not joined a sports club”, or “I have joined a sports club, but I do not participate in it”. We categorized responses based on participation in a sports club.

The dependent variables were weekly physical activity, perceived health status, and health behaviors. Weekly physical activity was assessed using the question, “How often do you participate in physical activity in a year?” The response options were “never”, “less than three days a month”, “one day a week”, “two days a week”, “three days a week”, “four days a week”, “five days a week”, “six days a week”, or “every day”. We categorized responses as “rarely”, “one–two days”, “three–four days”, or “five or more days”. Perceived health status was evaluated using the question “How healthy do you think you are right now?” The response options were “not at all healthy”, “not very healthy”, “moderately healthy”, “rather healthy”, or “very healthy”. We categorized responses as follows: “not at all healthy” and “not very healthy” as “poor”, “moderately healthy” as “average”, and “rather healthy” and “very healthy” as “good”.

The health behaviors examined were sufficient rest and sleep, as well as regular meals and nutritional supplementation. Whether one had sufficient rest and sleep was assessed using the question “How often do you take sufficient rest and sleep to maintain your health and physical fitness?” The response options were “never”, “rarely”, sometimes”, “often”, or “always”. We categorized responses as follows: “never” and “rarely” as “not performing”, “sometimes” as “neutral”, and “often” and “always” as “performing”. Whether one took regular meals and nutritional supplements was evaluated using the question “How often do you take regular meals and nutritional supplements to maintain your health and physical fitness?” The response options were “never”, “rarely”, “sometimes”, “often”, or “always”. We categorized responses as follows: “never” and “rarely” as “not performing”, “sometimes” as “neutral”, and “often” and “always” as “performing”. The response was modified to assess the association between the response and variable, rather than the sequence of the responses.

### 2.3. Covariate Variables

Covariate variables included sex, age, participation in lifestyle physical education classes, use of exercise prescription and consultation services, and success in quitting drinking and smoking. Sex was categorized as male or female. Age was categorized as being in one’s 60s, 70s, 80s, or older than 80. Participation in lifestyle physical education classes was determined using the question “Have you ever taken lifestyle physical education classes?” The response options were “yes” or “no”. Responses were used without any modifications. The use of exercise prescription and consultation services was determined using the question “Do you use exercise prescription and consultation services to manage your physical fitness?” The response options were “yes” or “no”. Responses were used without any modifications. Success in quitting drinking and smoking was identified using the question “How successful have you been in quitting drinking and smoking to maintain your health and physical fitness?” The response options were “not at all successful”, “not very successful”, “somewhat successful”, “a little successful”, or “very successful”. We categorized responses as follows: “not at all successful” and “not very successful” as “not performing”, “somewhat successful” as “neutral”, and “a little successful” and “very successful” as “performing”.

### 2.4. Data Analysis

The data were analyzed as follows: First, we conducted a frequency analysis to determine the characteristics of the study population. Second, we performed chi-square analyses to determine differences in older adults’ characteristics based on whether they participated in a sports club. Third, we performed multivariate logistic regression analyses to examine how participation in sports clubs is associated with weekly physical activity, perceived health status, and health behaviors. Thus, odds ratios (ORs) and 95% confidence intervals (CIs) were calculated. Since the chi-square test and logistic regression used different estimation methods, they can yield different results. If the chi-square test yielded statistically significant results, a logistic regression was added. All statistical analyses were performed using SPSS for Windows (version 23.0; IBM Corp., Armonk, NY, USA), with statistical significance set at *p* < 0.05.

## 3. Results

### 3.1. Characteristics of the Study Population

Most participants were female (52.4%), in their 60s (52.3%), and did not participate in sports clubs (90.7%) (Table 1). The largest group (38.0%) rarely participated in weekly physical activity. Many individuals perceived their health status as average (40.1%). Most individuals took sufficient rest and sleep (57.4%) and had regular meals and nutritional supplements (55.2%). Furthermore, most individuals did not use exercise prescription and consultation services (90.7%) or take lifestyle physical education classes (89.9%) but quit drinking and smoking (60.5%).

### 3.2. Differences in the Characteristics of the Study Population Based on Participation in Sports Clubs

Significant differences were found in sex (χ^2^ = 40.118, *p* < 0.001), age (χ^2^ = 39.146, *p* < 0.001), weekly physical activity (χ^2^ = 202.728, *p* < 0.001), perceived health status (χ^2^ = 121.093, *p* < 0.001), sufficient rest and sleep (χ^2^ = 28.248, *p* < 0.001), regular meals and nutritional supplementation (χ^2^ = 24.447, *p* < 0.001), use of exercise prescription and consultation services (χ^2^ = 253.140, *p* < 0.001), participation in lifestyle physical education classes (χ^2^ = 395.912, *p* < 0.001), and cessation of drinking and smoking (χ^2^ = 14.585, *p* = 0.001) (Table 2).

### 3.3. Association Between Participation in Sports Clubs and Weekly Physical Activity

Average ORs for weekly physical activity were 6.667 (95% CI = 4.316–10.297; *p* < 0.001) for 1–2 days, 5.237 (95% CI = 3.372–8.133; *p* < 0.001) for 3–4 days, and 3.042 (95% CI = 1.864–4.966; *p* < 0.001) for 5 or more days (Table 3).

### 3.4. Association Between Participation in Sports Clubs and Perceived Health Status

Average ORs for perceived health status were 1.601 (95% CI = 0.969–2.647; *p* = 0.066) and 2.780 (95% CI = 1.677–4.611; *p* < 0.001) for those who perceived their health status as average and good, respectively (Table 4).

### 3.5. Association Between Participation in Sports Clubs and Sufficient Rest and Sleep

Average ORs were 0.559 (95% CI = 0.264–1.183; *p* = 0.128) for insufficient rest and sleep and 1.272 (95% CI = 0.555–1.694; *p* = 0.100) for sufficient rest and sleep, respectively (Table 5).

### 3.6. Association Between Participation in Sports Clubs and Regular Meals and Nutritional Supplementation

Average ORs were 0.976 (95% CI = 0.497–1.915; *p* = 0.943) for irregular meals and nutritional supplementation and 1.028 (95% CI 0.770–1.373; *p* = 0.851) for regular meals and nutritional supplementation (Table 6).

## 4. Discussion

Our study yielded several insights. First, 38% of the study population rarely participated in physical activity, with only 9.3% participating in sports clubs. These results indicate that more than one-third of Korean older adults do not participate in physical activity. Physical activity among older adults is related to health management and improvements in quality of life. Therefore, it is necessary to explore ways to encourage older adults to participate in physical activities.

Second, participation in sports clubs increased the frequency of weekly physical activity, suggesting that sports clubs facilitate regular physical activities. This aligns with the results of previous studies showing that group-based participation in physical activity is more beneficial for older adults than solo participation [16,18,24,25,26]. Stolarz et al. [16] found that older adults who participate in sports clubs for more than five years engage in physical activities for a longer duration than those who participate in sports clubs for less than five years. The study showed a significant difference between the duration of physical activity and the active period in sports clubs. Moreover, a significant difference was detected between participation in sports clubs and weekly physical activity, as well as between the duration of sports club membership and the frequency of physical activity. These results suggest that the length of sports club membership affects the frequency of physical activity. Sports clubs encourage older adults to participate in physical activities, leading to regular participation in physical activities. Watts et al. [18] argued that sports clubs promote physical activity among older adults and reduce frailty. Participation of older individuals in sports club activities with local people encourages safe aging, facilitates socialization and learning, reduces difficulties in going out [24,25,26], and increases physical activity levels. Specifically, Kanamori et al. [24] found that group exercise may reduce the risk of physical and mental illness by improving adherence to physical activity, psychological well-being, and social relationships. Lin et al. [25] highlighted the cruciality of providing high-quality sports services tailored to the needs of different age groups within the elderly population. The growth of well-managed community sports organizations is essential in improving access to health and sports participation among elderly residents. The first senior center (William Hodson) opened in New York in 1943 and has since served as a “community focal point”, offering health, educational, social, and recreational services for one million older adults daily [26]. Therefore, participation in sports clubs can increase and maintain physical activity levels among older adults.

Third, participation in sports clubs improved the perception of health status among this population. This involved evaluating physical, physiological, psychological, and social aspects and focusing on wellness rather than disease. This perception can identify health conditions that cannot be determined by medical methods [27,28]. Our finding partially aligns with that of studies showing that older adults’ participation in physical activity positively affected their perceived health status [29,30,31]. Peralta et al. [30] examined the association between physical activity and self-rated well-being among European older adults in 28 countries. They found that physical activity in the last seven days was linearly related to overall self-rated well-being and its dimensions among both males and females, which is partially consistent with our finding. Promoting physical activity improves older adults’ well-being. Kim [31] found that perceived health status was positively related to quality of life among older adults who participated in physical activity. Meanwhile, Lindsay-Smith et al. [32] demonstrated that older adults’ participation in group physical activity with local residents improves their health-related quality of life, which is partially consistent with our findings. Overall, older adults must be encouraged to participate in sports clubs as they foster positive health perception.

Fourth, older adults’ participation in sports clubs was not significantly associated with taking sufficient rest and sleep. This result differs from that of previous studies [33,34,35]. Lee and Seo [33] found that the better the attitude of older adults toward participating in sports clubs, the more effectively participation in sports clubs improves their sleep disorders. These results may indicate that physical activity in the elderly is significantly associated with improved sleep quality. Kredlow et al. [34] conducted a meta-analysis of the effects of physical activity on sleep and found positive effects on sleep duration and efficiency, small-to-medium beneficial effects on sleep-onset latency, and moderately beneficial effects on sleep quality. Yang et al. [35] systematically reviewed the impact of physical activity on sleep quality among middle-aged and older adults with sleep disorders. Compared with the control group, the participants who were randomized to an exercise program had a better global Pittsburgh Sleep Quality Index score. Their results confirmed that physical activity is effective in treating individuals with sleep disorders. Considering that our finding differs from that of previous studies, further research is warranted.

Fifth, older adults’ participation in sports clubs was not significantly associated with taking regular meals and nutritional supplements. Other studies have shown that participation in physical activity helps maintain a balanced nutritional intake and prevent obesity [36,37,38]. Loprinzi et al. [37] demonstrated that physical activity is related to dietary behavior among American adults. Butterworth et al. [38] conducted a study among 30 older women with sedentary lifestyles, showing that the group that exercised 30–40 min a week for 12 weeks exhibited improved nutrient intake and meal quality. Aging, through peripheral and central mechanisms, reduces food intake and affects nutritional balance, potentially causing malnourishment [39,40]. Physical activity can benefit older adults by promoting balanced nutritional intake and healthy eating behaviors [41]. Considering that our result differs from that of previous studies, further research is needed.

## 5. Implications and Limitations

### 5.1. Implications and Recommendations

Physical activity can reduce the decline in physical function that occurs with aging. It can also prevent cognitive decline, increase independence, and alleviate depression, thereby improving the quality of life of older adults [42]. However, 38.0% of Korean older adults rarely participate in physical activity, with only 9.3% participating in sports clubs. We present the following suggestions for promoting physical activity and sports club participation among older adults. First, physical activity programs should be tailored to the health status of older adults. Programs should focus on aerobics, endurance, resistance, flexibility, mobility, and balance to achieve functional and health benefits among older adults [43]. Therefore, multidimensional physical activity programs tailored to older adults’ health status and characteristics may increase participation rates. Furthermore, sports instructors should be knowledgeable about the physical and psychological changes and diseases caused by aging to provide appropriate exercise guidance and handle emergencies. Thus, increasing expertise related to the sports activities of older adults is crucial [44]. In Korea, there are 55 sports activities for older adults; however, sports instructors are not trained for all of these sports, leading to a lack of expertise [45]. Regular training and field experience in each sport are necessary to increase their expertise [45], which will lead to the creation of tailored physical activity programs and the revitalization of physical activities among older adults.

Second, support should be provided to boost older adults’ participation in community-based sports clubs. Urban areas offer older adults easy access to sports centers. Conversely, rural areas face facility limitations and financial burdens [46]. Establishing sports centers and recreational programs for older adults in rural areas will provide them with opportunities to form sports clubs and participate in group physical activities. Communities can create swimming pools or fitness clubs to facilitate joint physical activities for older adults and other residents. Local governments can dispatch sports instructors to senior centers and offer free physical activity guidance. Draper et al. [47] showed that community-based physical activity programs can improve the health, quality of life, and physical activity awareness of older adults in underdeveloped areas. Local sports competitions can also increase older adults’ participation in physical activities. In Poland, some sports competitions require teams to include at least one older person, and recreational events award a prize to the oldest participant [48]. Additionally, Universities of the Third Age (U3A) offer education for older adults, specifically for those who are retired or in the “third age” of life [49]. U3A programs include sports, physical education, and art classes, where seniors participate in these programs to spend their leisure time and strengthen social interaction and networking [50]. Overall, U3A programs promote intellectual, mental, and physical activation among older adults, significantly impacting their quality of life in old age. Moreover, providing administrative and financial support can encourage older adults to participate in community-based sports clubs, improve their health and quality of life, and mitigate aging issues.

### 5.2. Limitations

The current study is highly representative and significant because it opens avenues for research on sports clubs for older adults. Nonetheless, this study had several limitations. First, the use of secondary data precluded the establishment of causal relationships before and after the survey. Second, data on perceived health status, sufficient rest and sleep, and regular meals and nutritional supplementation were based on subjective judgment rather than scientific measurements; the individual’s health status at the time of the survey did not affect the survey results. Participants might have overestimated their physical activity or perceived health status. Third, despite the anonymity guaranteed in the survey, the respondents may have exaggerated or underreported their health-related behaviors. Fourth, the results of this study cannot be generalized to all Koreans or elderly people worldwide because the study was conducted on 5146 elderly people and used data from elderly people aged 60 years or older among 18,000 Koreans aged 10 years or older. Furthermore, this study did not conduct analyses according to the sex and age of elderly people, which may have affected the results. Fifth, the use of basic statistical tests limited the depth of analysis because of the lack of a novel conceptual model based on current theories in aging activities and psychological aspects. Sixth, while multivariate logistic regression was useful in this study, it may not fully leverage the potential of a large dataset given the complexity of aging activities and psychological aspects. To strengthen the analysis, future studies should compensate for the current limitations by conducting research involving structural equation modeling or hierarchical linear modeling.

## 6. Conclusions

This study examined the association between participation in sports clubs and weekly physical activity, perceived health status, and health behaviors (sufficient rest and sleep and regular meals and nutritional supplementation) among Korean older adults. Older adults’ participation in sports clubs increased the frequency of weekly physical activity and improved their perceptions of their health status. These results suggest that participating in sports clubs can help older adults have healthier and more active lives. Participation in sports clubs should be emphasized to promote physical activity among older adults. Participation of the elderly in sports clubs may solve the challenges faced by Korea concerning the physical and mental health of this population. Therefore, policies focusing on the promotion of elderly people’s participation in sports clubs as part of elderly welfare should be established and implemented. Future research needs to explore the motivators of older adults to participate in sports clubs and provide them with these motivators. Additionally, qualitative studies are needed to confirm the in-depth experiences of elderly participants in sports clubs and reveal differences from the results of this quantitative research. Research is needed to develop strategies to promote older people’s participation in sports clubs. Additionally, elderly people in small provincial cities face infrastructure limitations that hinder their participation in sports clubs. Policy research is also needed to promote participation in sports clubs among elderly residents of small provincial cities.

## Figures and Tables

**Table 1:** Characteristics of the study population (n = 5146).

Characteristic	Categories	n/Total (%)
Sex	Male	2451/5146 (47.6%)
	Female	2695/5146 (52.4%)
Age, years	60s	2691/5146 (52.3%)
	70s	2341/5146 (45.5%)
	80s or older	114/5146 (2.2%)
Participation in sports clubs	Yes	481/5146 (9.3%)
	No	4665/5146 (90.7%)
Weekly physical activity	Rarely	1954/5146 (38.0%)
	1–2 days	1048/5146 (20.3%)
	3–4 days	1218/5146 (23.7%)
	5 or more days	926/5146 (18.0%)
Perceived health status	Poor	1059/5146 (20.6%)
	Average	2066/5146 (40.1%)
	Good	2021/5146 (39.3%)
Sufficient rest and sleep	Not performing	407/5146 (7.9%)
	Neutral	1785/5146 (34.7%)
	Performing	2954/5146 (57.4%)
Regular meals and nutritional supplementation	Not performing	369/5146 (7.2%)
	Neutral	1934/5146 (37.6%)
	Performing	2843/5146 (55.2%)
Use of exercise prescription and consultation services	Yes	481/5146 (9.3%)
	No	4665/5146 (90.7%)
Participation in lifestyle physical education classes	Yes	521/5146 (10.1%)
	No	4625/5146 (89.9%)
Cessation of drinking and smoking	Not performing	940/5146 (18.3%)
	Neutral	1091/5146 (21.2%)
	Performing	3115/5146 (60.5%)

**Table 2:** Differences in the characteristics of the study population based on participation in sports clubs.

Characteristic	Categories	Participation in Sports Clubs (n/Total [%])		χ^2^ (*p*)
		Yes	No	
Sex	Male	231/2451 (9.4%)	2220/2451 (90.6%)	40.118 (<0.001 ***)
	Female	132/2695 (4.9%)	2563/2695 (95.1%)	
Age, years	60s	246/2691 (9.1%)	2445/2691 (90.9%)	39.146 (<0.001 ***)
	70s	115/2341 (4.9%)	2226/2341 (95.1%)	
	80s or older	2/114 (1.8%)	112/114 (98.2%)	
Weekly physical activity	Rarely	27/1954 (1.4%)	1927/1954 (98.6%)	202.728 (<0.001 ***)
	1–2 days	140/1048 (13.4%)	908/1048 (86.6%)	
	3–4 days	143/1218 (11.7%)	1075/1218 (88.3%)	
	5 or more days	53/926 (5.7%)	873/926 (94.3%)	
Perceived health status	Poor	21/1059 (2.0%)	1038/1059 (98.0%)	121.093 (<0.001 ***)
	Average	105/2066 (5.1%)	1961/2066 (94.6%)	
	Good	237/2021 (11.7%)	1784/2021 (88.3%)	
Sufficient rest and sleep	Not performing	10/407 (2.5%)	397/407 (97.5%)	28.248 (<0.001 ***)
	Neutral	101/1785 (5.7%)	1684/1785 (94.3%)	
	Performing	252/2954 (8.5%)	2702/2954 (91.5%)	
Regular meals and nutritional supplementation	Not performing	13/369 (3.5%)	356/369 (96.5%)	24.447 (<0.001 ***)
	Neutral	106/1934 (5.5%)	1828/1934 (94.5%)	
	Performing	244/2843 (8.6%)	2599/2843 (91.4%)	
Use of exercise prescription and consultation services	Yes	119/481 (24.7%)	362/481 (75.3%)	253.140 (<0.001 ***)
	No	244/4665 (5.2%)	4421/4665 (94.8%)	
Participation in lifestyle physical education classes	Yes	147/521 (28.2%)	374/521 (71.8%)	395.912 (<0.001 ***)
	No	216/4625 (4.7%)	4409/4625 (95.3%)	
Cessation of drinking and smoking	Not performing	88/940 (9.4%)	852/940 (90.6%)	14.585 (0.001 **)
	Neutral	88/1091 (8.1%)	1003/1091 (91.9%)	
	Performing	187/3115 (6.0%)	2928/3115 (94.0%)	

** *p* < 0.01, *** *p* < 0.001; tested using chi-square analyses.

**Table 3:** Association between participation in sports club activities and weekly physical activity.

Variable		Participation in Sports Clubs, Odds Ratio (95% Confidence Interval)	*p*-Value
Weekly physical activity	Rarely	1.000	
	1–2 days	6.667 (4.316–10.297)	<0.001 ***
	3–4 days	5.237 (3.372–8.133)	<0.001 ***
	5 or more days	3.042 (1.864–4.966)	<0.001 ***

*** *p* < 0.001; assessed through multivariate logistic regression analysis by adjusting for sex, age, participation in lifestyle physical education classes, use of exercise prescription and consultation services, and cessation of drinking and smoking.

**Table 4:** Association between participation in sports clubs and perceived health status.

Variable		Participation in Sports Clubs, Odds Ratio (95% Confidence Interval)	*p*-Value
Perceived health status	Poor	1.000	
	Average	1.601 (0.969–2.647)	0.066
	Good	2.780 (1.677–4.611)	<0.001 ***

*** *p* < 0.001; assessed through multivariate logistic regression analysis by adjusting for sex, age, participation in lifestyle physical education classes, use of exercise prescription and consultation services, and cessation of drinking and smoking.

**Table 5:** Association between participation in sports clubs and sufficient rest and sleep.

Variable		Participation in Sports Clubs, Odds Ratio (95% Confidence Interval)	*p*-Value
Sufficient rest and sleep	Neutral	1.000	
	Not performing	0.559 (0.264–1.183)	0.128
	Performing	1.272 (0.555–1.694)	0.100

Assessed through multivariate logistic regression analysis by adjusting for sex, age, participation in lifestyle physical education classes, use of exercise prescription and consultation services, and cessation of drinking and smoking.

**Table 6:** Association between participation in sports clubs and regular meals and nutritional supplementation.

Variable		Participation in Sports Clubs, Odds Ratio (95% Confidence Interval)	*p*-Value
Regular meals and nutritional supplementation	Neutral	1.000	
	Not performing	0.976 (0.497–1.915)	0.943
	Performing	1.028 (0.770–1.373)	0.851

Assessed through multivariate logistic regression analysis by adjusting for sex, age, participation in lifestyle physical education classes, use of exercise prescription and consultation services, and cessation of drinking and smoking.

## Data Availability

The data that support the findings of this study are available from the corresponding author(s) upon reasonable request.
